# Carboxytherapy in Androgenetic Alopecia: Hype or Hope?

**DOI:** 10.1111/jocd.71059

**Published:** 2026-07-12

**Authors:** Fatemeh Mokhtari, Hamzeh Shafiei, Gita Faghihi, Azin Mohamadsalehi

**Affiliations:** ^1^ Department of Dermatology, School of Medicine, Skin Diseases and Leishmaniasis Research Center Isfahan University of Medical Sciences Isfahan Iran

**Keywords:** androgenetic alopecia, Carboxytherapy, finasteride, minoxidil

## Abstract

**Background:**

Androgenetic alopecia (AGA) is the most common type of hair loss in men and can impose a significant psychosocial burden, reducing self‐esteem and quality of life. Current standard treatments include topical minoxidil and oral finasteride; however, there is no definitive or fully satisfactory treatment. Carboxytherapy increases blood flow and enhances the delivery of oxygen and nutrients to hair follicles. It may also facilitate transdermal drug delivery and improve the response to standard therapy.

**Methods:**

Forty‐six male patients aged 18–50 years with moderate‐to‐severe androgenetic alopecia (AGA) were allocated into two groups. The intervention group received topical minoxidil 5%, oral finasteride 1 mg, and carboxytherapy. A total of six sessions were administered at 2‐week intervals over approximately 12 weeks. The control group received topical minoxidil 5% and oral finasteride 1 mg alone. Outcomes were assessed at baseline, 3 months, and 24 weeks post‐treatment using standardized photography, dermoscopy, and patient satisfaction based on a 7‐point grading scale.

**Results:**

At 24 weeks post‐treatment, both groups showed statistically significant improvements in patient satisfaction and photography‐dermoscopy scores. However, the intervention group achieved significantly higher dermoscopy‐photography scores than the control group (*p* = 0.006).

**Conclusion:**

Adding carboxytherapy to standard therapy (topical minoxidil and oral finasteride) resulted in greater clinical improvement and higher patient satisfaction compared with standard therapy alone.

## 1 Introduction

Androgenetic alopecia (AGA) is the most common form of progressive hair loss in men. It is more prevalent among Caucasians, affecting approximately 50% of men by the age of 50 and up to 80% of men during their lifetime, as well as about 50% of women. AGA results from the miniaturization of terminal hairs into fine vellus hairs in genetically predisposed individuals [1, 2, 3]. Beyond its dermatologic manifestations, AGA can impose a significant psychosocial burden, reducing self‐esteem and quality of life in affected individuals [4]. AGA involves androgen (particularly dihydrotestosterone [DHT])‐mediated miniaturization of hair follicles and shortening of the anagen phase [1, 5].

Current standard treatments include topical minoxidil, which promotes vasodilation and prolongs the anagen phase via upregulation of growth factors such as vascular endothelial growth factor (VEGF), and oral finasteride, a type I that lowers intrafollicular DHT and slows miniaturization [3, 5, 6, 7]. Despite proven benefits compared with placebo, therapeutic responses are often incomplete and vary among individuals. In addition, the need for long‐term use and potential adverse effects may lead some patients to discontinue treatment. Therefore, combination therapy may provide better outcomes.

In recent years, several attractive therapeutic modalities have been investigated as adjuncts or alternatives to standard therapy in AGA. For example, recent studies have demonstrated the efficacy of platelet‐rich plasma (PRP) in treating androgenetic alopecia. Shah et al. [8], reported that PRP significantly improves hair density in men with AGA [8].

Fractional and non‐ablative lasers, including 1540‐nm erbium‐glass systems, induce controlled dermal remodeling, increase local perfusion, and may enhance permeability to topical agents. Mokhtari et al. demonstrated that combining an erbium:YAG laser with 5% minoxidil is more effective than minoxidil alone in treating AGA [9]. Despite these emerging and attractive modalities, clinical responses remain heterogeneous, and combination strategies are increasingly recommended to optimize therapeutic outcomes in AGA.

Carboxytherapy is hypothesized to act through rapid modulation of local tissue perfusion and oxygen delivery. Intradermal CO_2_ induces rapid local vasodilation and augments microcirculation, increasing blood flow and the delivery of oxygen and nutrients to hair follicles. In addition, diffusion of CO_2_ into tissue shifts the oxyhemoglobin dissociation curve (Bohr effect), enhancing oxygen off‐loading in the follicular microenvironment and thereby improving follicular metabolism [10, 11, 12, 13]. When combined with minoxidil and finasteride, the overall effect may be synergistic, targeting vascular, metabolic, and androgenic contributors to follicular miniaturization [14].

CO_2_ exposure may also activate dermal fibroblasts and promote remodeling of the extracellular matrix, including increased collagen and elastin synthesis. Improved dermal structural integrity and thickness may provide a more supportive niche for hair follicles and indirectly improve hair shaft caliber and visible density [12, 14].

Furthermore, carboxytherapy may facilitate transdermal drug delivery.

Intradermal CO_2_ injection can create microchannels in the dermis, temporarily increasing skin permeability. These microchannels may enhance the penetration and efficacy of concurrently applied topical agents, such as minoxidil. This dual mechanism may contribute to the synergistic effect observed when carboxytherapy is combined with standard pharmacologic therapies [10, 12]. Although carboxytherapy is widely applied in aesthetic fields (e.g., skin rejuvenation and scar remodeling), its clinical utility in alopecia, particularly AGA, remains under investigated [10, 15].

Preliminary clinical studies have reported beneficial effects of carboxytherapy on hair density and patient satisfaction; however, they are limited by small sample sizes, heterogeneous methodologies, and short follow‐up periods. Moreover, a few trials in female pattern hair loss have suggested that combining carboxytherapy with minoxidil yields greater improvement than minoxidil alone [11]. Systematic reviews of carboxytherapy in dermatology emphasize its potential but call for rigorously designed randomized controlled trials to clarify efficacy, optimal dosing regimens, and durability of response [16, 17, 18]. Bagherani et al. highlighted potential mechanisms of CO_2_ therapy, including enhanced microcirculation, angiogenesis, and extracellular matrix remodeling, supporting its possible role in hair restoration [12]. Mechanistic studies by Koutna et al. suggested that local CO_2_ application enhances follicular oxygenation and capillary perfusion, promoting anagen induction [19].

To our knowledge, no well‐powered randomized trial has assessed the triple combination of intradermal CO_2_, minoxidil, and finasteride specifically in male AGA.

Therefore, this controlled clinical study aimed to evaluate whether adding intradermal carboxytherapy to standard therapy (minoxidil + finasteride) yields superior outcomes in hair growth and patient satisfaction compared with standard therapy alone in men with AGA.

## 2 Methods and Participants

This randomized, parallel‐group trial was conducted on 46 male patients with moderate to severe AGA who were referred to medical centers medical centers affiliated with Isfahan University of Medical Sciences from October 2023 to January 2025. (IR.MUI.MED.REC.1403.056)(IRCT20241005063265N1).

### 2.1 Patient

The inclusion criteria were men aged 18–50 years with Hamilton–Norwood stages II–V who had not received minoxidil, finasteride, or any other hair growth intervention within the preceding 24 weeks (Table 1). Exclusion criteria included prior hair transplantation, scalp infections, or other dermatologic disorders affecting the scalp (such as alopecia areata, telogen effluvium, anagen effluvium, and scarring alopecias), as well as serious systemic disease.

**TABLE 1:** Demographic and clinical characteristics of the patients.

Characteristics	finasteride + minoxidil	Combination carboxytherapy and finasteride + minoxidl	T	P_Value
Age	31.10 ± 8	32.23 ± 7	0.509	0.613
Duration of disease	5.3 ± 4	6.4 ± 5	0.824	0.415
Grade of Hair loss	4.1 ± 1	3.9 ± 1	0.678	0.501

### 2.2 Randomization

**FIGURE 1:**
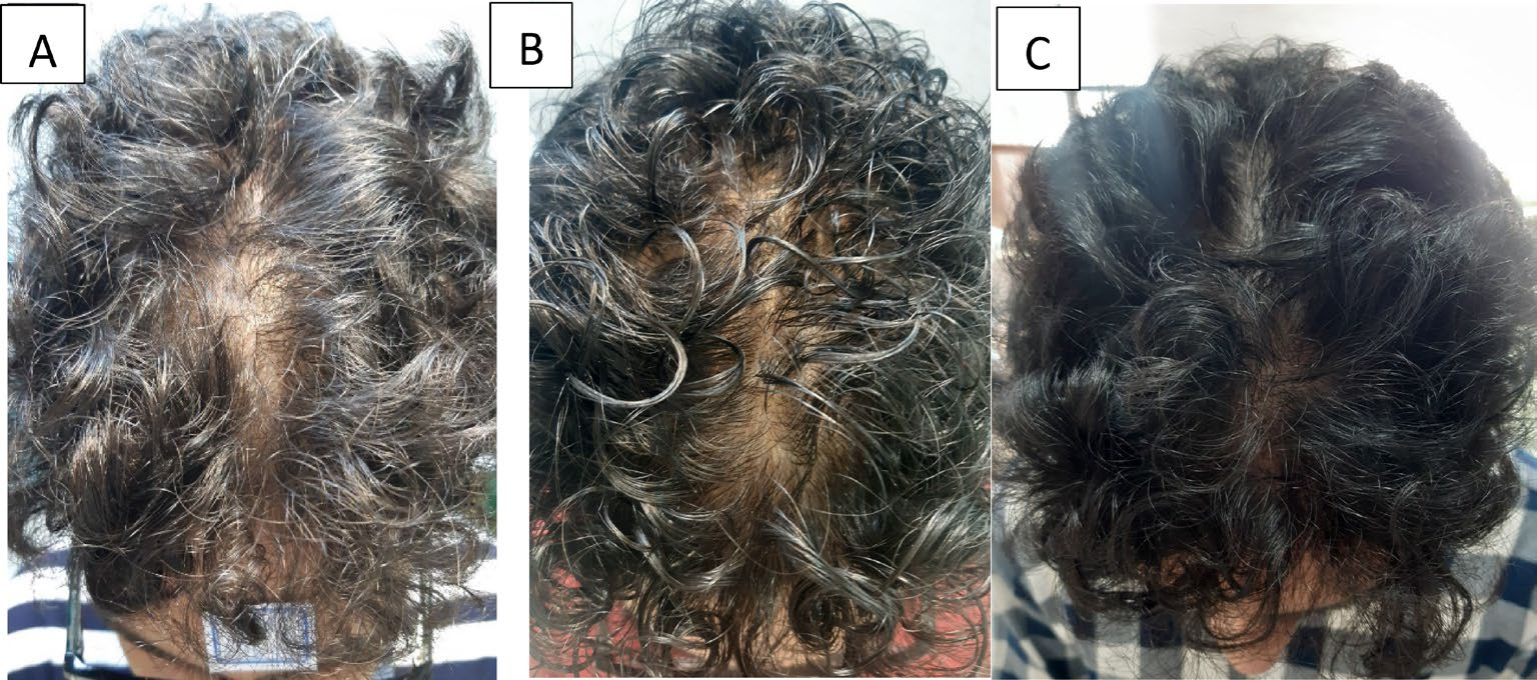
Week 0 (A), week 12 (B), and week 24 (C) clinical appearance in a patient receiving carboxytherapy in combination with topical minoxidil and oral finasteride.

**FIGURE 2:**
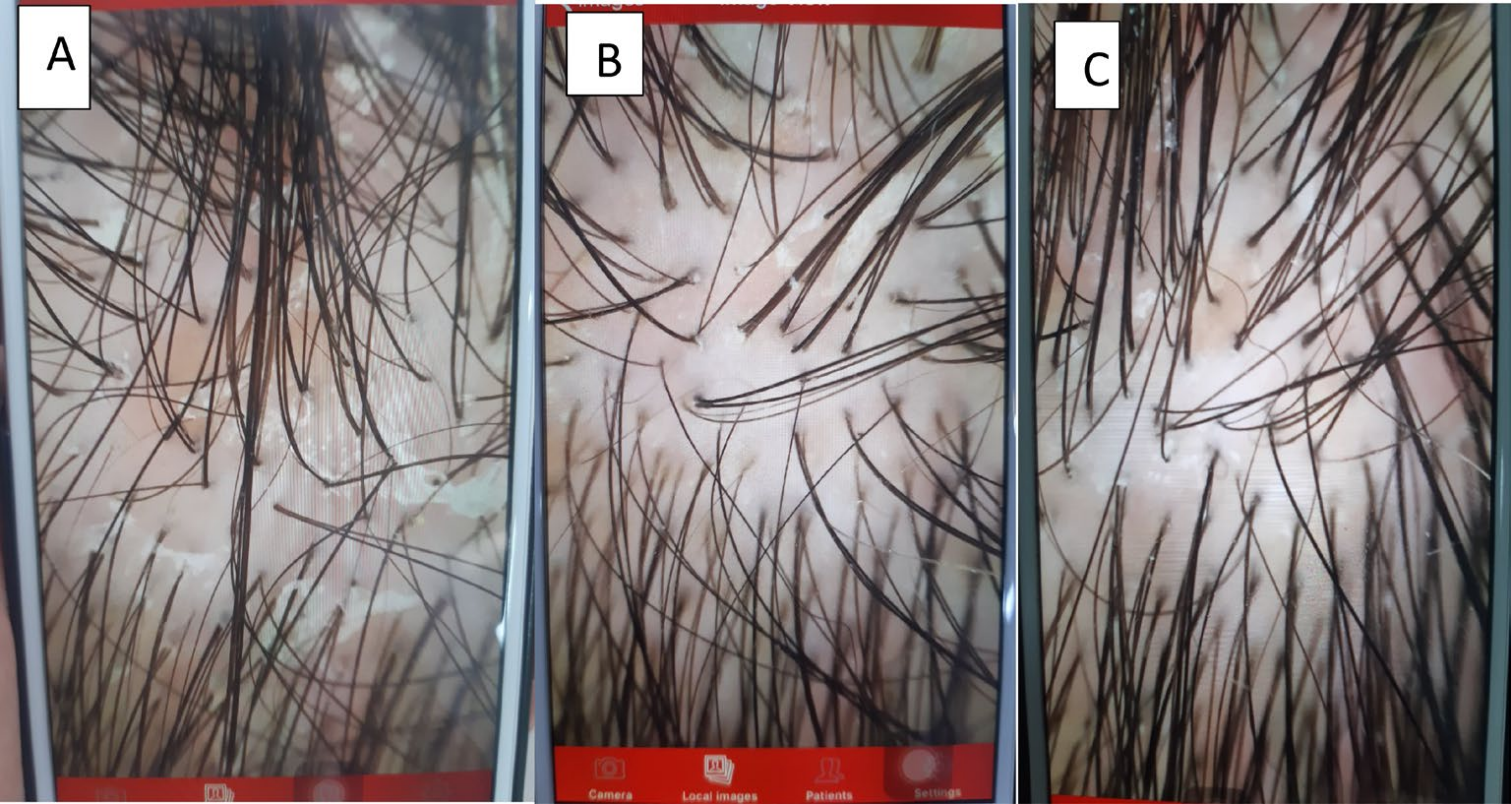
Week 0 (A), week 12 (B), and week 24 (C) dermoscopic images of the same scalp point in a patient receiving carboxytherapy in combination with topical minoxidil and oral finasteride.

A total of 46 eligible patients were sequentially numbered from 1 to 46 and then randomly allocated in a 1:1 ratio to either the intervention or control group using a computer‐generated random sequence. Randomization was performed by an investigator who was not involved in patient recruitment or outcome assessment to reduce allocation bias Figures 1, 2.

### 2.3 Interventions

**FIGURE 3:**
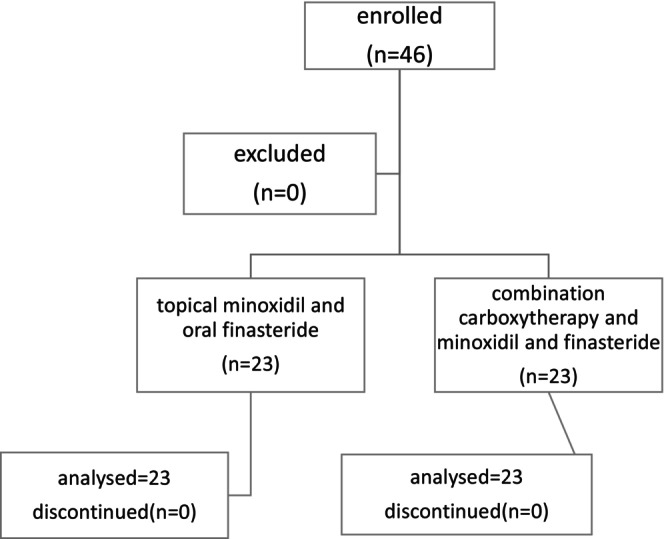
CONSORT flow diagram of patient enrollment and allocation.

This was a single‐blind, parallel‐group randomized controlled trial. Both groups received standard therapy consisting of topical 5% minoxidil (Pak Daru, Iran) applied twice daily and oral finasteride 1 mg (Finoscar, Atipharmed, Iran) taken once daily. The intervention group additionally received intradermal carboxytherapy sessions Figure 3.

### 2.4 Carboxytherapy Procedure

A topical anesthetic cream, a mixture of 2.5% lidocaine/prilocaine (Xyla‐P, Tehran Chemie Pharmaceutical Company, Iran), was applied to the area to be treated 30–45 min prior to carboxytherapy procedure. The area was then washed with saline and cleansed with betadine.

For Carboxytherapy procedure we used[device Ocotillo/Exon medical technologies‐Iran], injecting medical‐grade CO_2_ intradermally in affected scalp areas. A total of six sessions were administered at two‐week intervals over approximately 12 weeks. In each session, 50–80 mL of CO_2_ was injected at a controlled pressure of 1.5–2.0 bar (flow rate: 1 cc/s). Injections were administered using a 30G needle (length: 4–6 mm) at a depth of 1–2 mm. The gas volume per injection point was 2 mL, delivered over approximately 1–2 s. Injection sites were spaced 1–1.5 cm apart, covering the frontal and vertex regions. After the procedure, the scalp was cleaned with gauze moistened with saline, and patients were instructed not to apply minoxidil on the day of the procedure. Total procedure time per session was approximately 10–15 min. All procedures were performed by a trained dermatologist under standardized aseptic conditions.

### 2.5 Assessments

Demographic information was recorded for all patients. Disease severity was evaluated using the Hamilton–Norwood scale before initiation of treatment. Safety and adverse effects, including symptoms of infection, prolonged erythema, burning, and itching of the scalp, were assessed at each visit.

Treatment outcomes were evaluated using two methods: (1) photography–dermoscopy and (2) patient satisfaction assessment, performed 24 weeks after the start of treatment.

Standardized high‐resolution scalp photographs were obtained before treatment and at the end of the 6‐month treatment period. Photographs were taken in four vertex views, covering the mid‐scalp, temporal, and frontal regions.

Dermoscopic evaluation was performed using the FotoFinder Handyscope (FotoFinder Systems GmbH, Bad Birnbach, Germany) at three marked points on the scalp located 12, 16, and 24 cm from the glabella, corresponding to the frontal, mid‐scalp, and vertex areas. Dermoscopic photographs were taken from the center of each marked point.

Photography–dermoscopy results were graded using a 7‐point scale:

Greatly decreased = −3.

Relatively decreased = −2.

Slightly decreased = −1.

No change = 0.

Slightly increased = +1.

Proportionally increased = +2.

Greatly increased = +3.

Two dermatologists blind to group allocation independently evaluated the clinical and dermoscopic images and assigned a single combined 7‐point score per patient. In cases of disagreement, the evaluators discussed the case and reached a consensus score, which was recorded as the final score.

### 2.6 Patient Satisfaction

Patient‐perceived treatment satisfaction was evaluated at the end of 24 weeks using a 7‐point ordinal scale:

−3 = significant deterioration.

−2 = moderate deterioration.

−1 = slight deterioration.

0 = no change.

+1 = slight improvement.

+2 = moderate improvement.

+3 = significant improvement.

Assessments were performed at baseline (week 0), after completion of the sixth carboxytherapy session (12 weeks), and at 24 weeks. Standardized scalp photographs and dermoscopic images were obtained under identical lighting and positioning conditions. Adherence to minoxidil and finasteride was monitored using patient diaries and medication counts at monthly visits.

### 2.7 Statistical Analysis

Statistical analyses were performed using IBM SPSS Statistics version 16 (IBM Corp., Armonk, NY, USA). Normality of data distribution was assessed using the Shapiro–Wilk test. As the data were approximately normally distributed, comparisons between the carboxytherapy and control groups were conducted using independent‐samples *t*‐tests.

Mean and standard deviation (SD) values were calculated for each variable. Effect sizes were estimated using Cohen's d to assess the magnitude of between‐group differences. A two‐tailed *p* value < 0.05 was considered statistically significant.

## 3 Results

A total of 46 patients were included in this study, with a mean age of 31.7 ± 7.5 years. The mean duration of hair loss was 5.85 ± 4.5 years. The two groups were comparable in their demographic and clinical characteristics, including age, disease duration, and grade of hair loss.

Patients in both groups showed a favorable response to treatment and achieved statistically significant improvement as evaluated by patient satisfaction and photography–dermoscopy outcomes Figures 1, 2.

The dermoscopic–photographic evaluation demonstrated a significant difference between the groups after 24 weeks. The intervention group achieved a higher dermoscopy–photography score than the control group (Table 2). In addition, patient satisfaction was significantly higher in the combination therapy group compared with the control group (*p* < 0.05) (Table 3).

**TABLE 2:** Improvement in dermatologist scores in the two groups at 24 weeks post‐treatment.

Group	N	Mean ± SD	T	P_Value	Cohen's d
Intervention	23	1.43 ± 0.94	2.899	0.006	0.855
Control	23	0.66 ± 0.885			

**TABLE 3:** Improvement in patient satisfaction scores in the two groups at 24 weeks post‐treatment.

Group	N	Mean ± SD	t	P_Value	Cohen's d
Intervention	23	1.7 ± 1.15	2.879	0.006	0.849
Control	23	0.83 ± 0.89			

### 3.1 Side Effects

The most common adverse effects were erythema and itching resembling seborrheic dermatitis, which occurred in 6 patients in the intervention group and 4 patients in the control group.

Pain during the carboxytherapy procedure was also reported. Five patients experienced mild pain and slight burning during the procedure. These symptoms were transient, resolved spontaneously, and did not require additional intervention.

**FIGURE 4:**
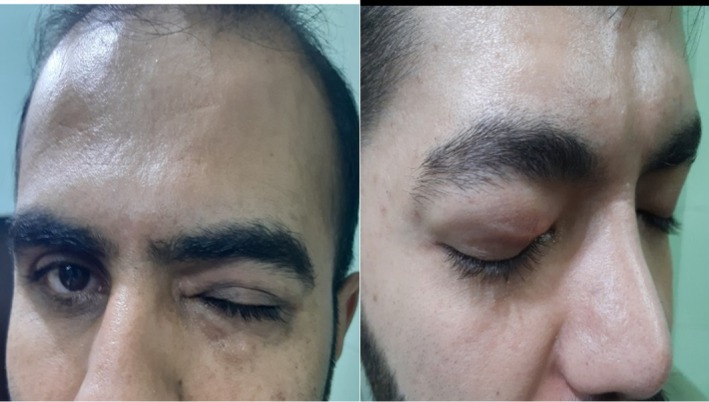
Periorbital edema due to subcutaneous gas accumulation around the eyes.

Additionally, two patients developed transient periorbital edema (Figure 4) due to subcutaneous gas accumulation around the eyes. The edema resolved within 48–72 h. Patients were advised to perform gentle massage of the periorbital area and to keep their heads elevated during rest to facilitate faster absorption of the gas.

Evidence suggests that carboxytherapy is generally well tolerated, with no serious long‐term adverse effects reported to date. Reported complications are usually mild and self‐limited. In our study, no serious or persistent long‐term adverse effects were observed during the 6‐month follow‐up period.

## 4 Discussion

Androgenetic alopecia (AGA) is a common progressive hair loss, causing many cosmetic problems for men and women worldwide and covers up to 80% of males by the age of 80 [20], and based on its prevalence, different treatments have been proposed to stop the progression of hair loss and stimulate the regrowth of hair [3].

There are only two FDA‐approved medications to treat AGA: topical minoxidil and systemic finasteride [2, 5, 21]. Recently, carboxytherapy has been introduced as an adjuvant therapy for hair regrowth in AGA. Our results showed that adding carboxytherapy to a standard regimen of topical minoxidil and oral finasteride produced significantly greater clinical improvement in androgenetic alopecia over a 6‐month period compared with minoxidil and finasteride alone.

Carboxytherapy, involving controlled intradermal or subcutaneous injections of carbon dioxide (CO_2_), has gained interest as an adjunctive dermatologic modality.

Its hypothesized mechanisms relevant to hair regrowth include induction of local vasodilation, enhanced tissue perfusion, upregulation of angiogenic mediators (e.g., VEGF), and promotion of oxygen release to tissues via the Bohr effect [15, 16].

These physiologic alterations may provide a more favorable microenvironment for hair follicle metabolism and activation of dormant follicles.

Several studies have demonstrated the potential efficacy of carboxytherapy in promoting hair growth. Doghaim et al. conducted a prospective controlled trial on 80 patients, including 40 cases of androgenetic alopecia and 40 cases of alopecia areata. In each subgroup, 20 patients received intradermal CO_2_ injections while the other 20 were treated with placebo (distilled water injections) for six weekly treatment sessions. Their study demonstrated significant clinical improvement; however, follow‐up was limited to 3 months, and injections were administered at only 7 predefined points on the scalp [10].

In contrast, our trial exclusively focused on androgenetic alopecia with a more specialized design, enrolling 46 male patients who were randomized into two equal groups of 23 participants. Additionally, carboxytherapy in our study was performed alongside established standard therapy (topical minoxidil and oral finasteride), providing a combination approach rather than monotherapy. Sessions were conducted biweekly over 12 weeks, and CO_2_ injections were delivered in a diffuse pattern with 1–1.5 cm spacing across the affected frontal and vertex regions, ensuring more homogeneous scalp coverage. Furthermore, our follow‐up duration was extended to 24 weeks, allowing assessment of more durable treatment outcomes. Nilforooshzadeh et al. evaluated the efficacy of combining carboxytherapy with high‐concentration topical minoxidil (20%) and microneedling in a small group of nine patients with treatment‐resistant androgenetic alopecia. Their protocol included four monthly sessions in which carboxytherapy was immediately followed by minoxidil (20%) and microneedling. Clinical improvement was assessed at baseline and 3 months after the first treatment session. Although their results suggested that carboxytherapy may potentiate the effects of minoxidil in refractory cases, the very limited sample size, inclusion of only resistant AGA, and short follow‐up duration restrict the generalizability of their findings [11].

In comparison, our study investigated a broader AGA population, including patients with Hamilton‐Norwood grades II‐V rather than only resistant disease, and involved a substantially larger sample size with random allocation into two balanced groups of 23 patients each. Moreover, we applied carboxytherapy as part of a standardized combination regimen with topical 5% minoxidil and oral finasteride, reflecting real‐world therapeutic practice. Treatment sessions were performed biweekly, resulting in six sessions over approximately 12 weeks, and treatment efficacy was evaluated through blind assessment at 24 weeks follow‐up. Metwally et al. studied 30 patients with alopecia areata by comparing three treatment regions within each patient: intralesional corticosteroid injections alone, carboxytherapy alone, and a combination of both modalities. They observed that the combined‐treatment regions demonstrated higher clinical improvement compared with either treatment alone, supporting a potential synergistic role for carboxytherapy when used as an adjunct to established therapies. Their sessions were also performed biweekly, similar to our protocol; however, their work was limited by a relatively small sample size, lack of randomization, and evaluation restricted to alopecia areata [22].

In contrast, our study specifically targeted androgenetic alopecia and implemented a randomized controlled design with a larger sample size of 46 participants divided equally into two parallel groups. we combined carboxytherapy with standard therapy (topical 5% minoxidil + oral finasteride), enabling evaluation of its additive benefit in the real‐world management of male AGA. As discussed previously, these factors led us to design a study with these specifications. This design allowed us to assess not only the absolute efficacy of carboxytherapy but also its additive effect over established treatments.

Additionally, our evaluation included both objective measures (photographic–dermoscopic assessment) and patient‐reported outcomes, providing a comprehensive assessment of treatment efficacy and tolerability.

With regard to side effects periorbital edema due to subcutaneous gas accumulation: Based on procedural experience and prior reports, orienting the needle bevel upward during injection and applying gentle post‐procedural massage can reduce the likelihood of gas migration and subsequent edema.

Additionally, to control this side effect, we placed a headband on patients during the procedure to prevent gas from migrating toward the eyes.

Pain during the carboxytherapy procedure could be a limiting factor. These symptoms resolved by pre‐procedural topical anesthetic application (e.g., lidocaine) approximately 30–45 min prior to treatment, by performing slow injections, and by limiting the depth of needle penetration.

## 5 Conclusion

This study shows the addition of carboxytherapy to standard therapy (topical minoxidil and oral finasteride) resulted in greater clinical improvement and higher patient satisfaction compared with standard therapy alone. The procedure was generally well tolerated, with only transient and self‐limited adverse effects. These findings suggest that carboxytherapy may serve as a useful adjunct in the management of AGA.

### 5.1 Limitation

One important limitation of our study was the relatively small sample size. This was mostly because carboxytherapy was less known and newer than other methods such as PRP, etc., and patients were less willing to participate in the study; the lack of objective assessments, such as hair density and thickness measurement, was another limitation of our study.

## Author Contributions

Fatemeh Mokhtari was responsible for conceptualization, carboxytherapy consult, patient management, data arrangement, drafting the manuscript and study design. Hamzeh Shafiei was responsible for patient management and drafting the manuscript and data arrangement. Gita Faghihi was responsible for carboxytherapy consult and data arrangement. Azin Mohamadsalehi was responsible for patient management and data arrangement.

## Ethics Statement

The study was approved by the Institutional Review Board and Ethics Committee of Isfahan University of Medical Sciences (ir.mui.MED.REC.1403.056).

## Consent

Written informed consent was obtained from all participants before enrollment in the study. All participants were informed about the study objectives, procedures, potential benefits, and possible risks. Clinical photographs were taken solely for scientific and publication purposes. Identifiable facial features were avoided, and patient anonymity was strictly maintained.

## Conflicts of Interest

The authors declare no conflicts of interest.

## Data Availability

The data supporting the findings of this study are not publicly available due to privacy and ethical restrictions related to patient confidentiality. However, the data are available from the corresponding author upon reasonable request and with permission from the Ethics Committee of Isfahan University of Medical Sciences.
